# Identification of Cilia in Different Mouse Tissues

**DOI:** 10.3390/cells10071623

**Published:** 2021-06-29

**Authors:** Xinhua Li, Shuting Yang, Vishwa Deepk, Zahra Chinipardaz, Shuying Yang

**Affiliations:** 1 Department of Basic and Translational Sciences, School of Dental Medicine, University of Pennsylvania, Philadelphia, PA 19104, USA; xinhuali1013@163.com (X.L.); yshut@upenn.edu (S.Y.); deepakv@upenn.edu (V.D.); zahrach@upenn.edu (Z.C.); 2 Department of Orthopedics, Shanghai General Hospital, Shanghai Jiao Tong University School of Medicine, Shanghai 200080, China; 3 Department of Spinal Surgery, East Hospital, School of Medicine, Tongji University, Shanghai 200120, China; 4 The Penn Center for Musculoskeletal Disorders, School of Medicine, University of Pennsylvania, Philadelphia, PA 19104, USA; 5 Center for Innovation & Precision Dentistry, School of Dental Medicine, School of Engineering and Applied Sciences, University of Pennsylvania, Philadelphia, PA 19104, USA

**Keywords:** primary cilia, centrin, ARL13B, nucleus pulposus, brain

## Abstract

Cilia are microtubule-based hair-like organelles that extend from the cell surface. However, the existence and distribution of cilia in each organ and tissue at the postnatal stage in vivo remain largely unknown. In this study, we defined cilia distribution and arrangement and measured the ciliary lengths and the percentage of ciliated cells in different organs and tissues in vivo by using cilium dual reporter-expressing transgenic mice. Cilia were identified by the presence of ARL13B with an mCherry+ signal, and the cilium basal body was identified by the presence of Centrin2 with a GFP+ signal. Here, we provide in vivo evidence that chondrocytes and cells throughout bones have cilia. Most importantly, we reveal that: 1. primary cilia are present in hepatocytes; 2. no cilia but many centrioles are distributed on the apical cell surface in the gallbladder, intestine, and thyroid epithelia; 3. cilia on the cerebral cortex are well oriented, pointing to the center of the brain; 4. ARL13B+ inclusion is evident in the thyroid and islets of Langerhans; and 5. approximately 2% of cilia show irregular movement in nucleus pulposus extracellular fluid. This study reveals the existence and distribution of cilia and centrioles in different tissues and organs, and provides new insights for further comprehensive study of ciliary function in these organs and tissues.

## 1. Introduction

Cilia are microtubule-based organelles that were first discovered by Leeuwenhoek in approximately 1675. Most types of cells only possess one cilium, named a primary cilium or monocilium, but some cells possess ciliary bundles that consist of 200–300 cilia per cell, named motile cilia, that enable shift of fluids or mucus along the surface of the ciliated cells. Primary cilium is usually immotile which has 9 + 0 axonemal structure, with nine outer microtubule doublets, while motile cilia are motile with 9 + 2 axonemal structure consisting of nine outer microtubule doublets and two centrally-located singlet microtubules. Basal body forms the base of the cilium and arises from the mother centriole of the centrosome. Most proximal part of cilium is so-called transition zone of unique ultrastructural organization. Although cilia have been investigated for many years, not all cell types or tissues have been studied for the presence of cilia, and how cilia are distributed and arranged in different organs and tissues in vivo remains largely unknown. To further identify cells with cilia, we tested for the presence of cilia and characterized the distribution of cilia in different organs and tissues by using a unique cilium double-reporter transgenic mouse model [1]. In this model, the ciliary protein ARL13B is fused with the monomeric red fluorescent protein mCherry, and the centriolar protein Centrin2 is fused with GFP to mark the cilium basal body. ARL13B was originally identified in genetic screenings as a protein essential for ciliated organ integrity and neural tube patterning [2]. It is a ciliary small GTPase protein that is located on cilia [3] and, therefore, has been used as a cilium marker in various mono- and multi-ciliated tissues [1,3]. In humans, mutations of ARL13B cause Joubert syndrome, a ciliopathy characterized by brain malformations, combined with polydactyly and renal cyst formation [4,5]. Centrioles are essential for spatially precise execution of cell division [6]. The ARL13B-mCherry;Centrin2-GFP cilium dual-reporter transgenic mouse line has been proven to exhibit successful labeling of cilia with mCherry and labeling of basal bodies with GFP, and the mice are viable and fertile without disruption of normal ciliary function [1].

Here, by using this unique cilium dual reporter-expressing transgenic mouse model, we analyzed the existence and distribution of cilia and centrioles in different tissues and organ systems. Our findings provide the first evidence on this topic and lay a foundation for further comprehensive study of ciliary function in the organs and tissues analyzed in this study.

## 2. Materials and Methods

### 2.1. Mice

All procedures for housing and breeding animals and collecting animal tissue were performed following the animal protocols approved by the Institutional Animal Care and Use Committee (IACUC) of the University of Pennsylvania in accordance with the IACUC’s relevant guidelines and regulations. ARL13B-mCherry; Centrin2-GFP cilium dual reporter-expressing transgenic mice [1] were purchased from Jackson Laboratory (Stock No: 027967, Bar Harbor, ME, USA). The ARL13B-mCherry; Centrin 2-GFP mice were generated from mice with a mixed background of FVB and C3H strains. Four-week-old male ARL13B-mCherry; Centrin2-GFP mice were used for this study.

### 2.2. Genotyping

Genomic DNA was isolated from tail tips by proteinase K digestion and extracted with a NucleoSpin tissue kit (Macherey-Nagel) according to the manufacturer’s instructions. PCR was performed using primers for ARL13B-mCherry (5-CTA GGC CAC AGA ATT GAA AGA TCT-3, 5-GTA GGT GGA AAT TCT AGC ATC ATC C-3) and Centrin2-GFP (5-TGA ACG AAA TCT TCC CAG TTT CA-3, 5-ACT TCA AGA TCC GCC ACA ACA T-3).

### 2.3. Preparation of Frozen Sections and Observation of Cilia

Mouse organs or soft tissues from 4-week-old ARL13B-mCherry; Centrin2-GFP cilium dual reporter-expressing transgenic mice (*n* = 6) were fixed with 10% neutral buffered formalin for 1–2 h at 4 °C [7,8]. Mouse femurs and intervertebral discs from between the third and fifth vertebrae in the lumbar spine and between the fifth and sixth coccygeal vertebrae (*n* = 6) were excised, fixed with 10% neutral buffered formalin, and decalcified in 10% ethylenediaminetetraacetic acid (EDTA) for 2–3 weeks at 4 °C. All harvested tissues were embedded in optimum cutting temperature (OCT) compound and sectioned using a standard microtome (Cryostat 29 cryotome, CM1950, Leica), and 6-μm sections were prepared. The coverslips were mounted with Fluoroshield (F6057, Sigma–Aldrich, St. Louis, MO, USA).

### 2.4. Immunofluorescence Staining

Tissue sections with a thickness of 6 μm were incubated with proteinase K (20 μg/mL, D3001-2-5, Zymo Research) for 10 min at room temperature. Subsequently, the sections were blocked in 5% normal goat serum for 1 h (10000C, Thermo Fisher Scientific, Swedesboro, NJ, USA) in PBS-T (0.4% Triton X-100 in PBS) and incubated with antibodies against acetylated tubulin (1:100, T6793, Sigma, St. Louis, MO, USA) or ARL13B (1:100, 17711-1-AP, Proteintech, Rosemont, IL, USA) in blocking buffer at 4 °C overnight. The tissue sections were washed 3 times with PBS. The tissues were incubated with Alexa Fluor 647-conjugated anti-mouse (1:200, A-21236, Invitrogen, Carlsbad, CA, USA) or anti-rabbit (1:200, sc-516251, Santa Cruz, CA, USA) secondary antibodies at 4 °C for 1 h. The coverslips were mounted with Fluoroshield (F6057, Sigma–Aldrich, St. Louis, MO, USA).

### 2.5. Quantitative Analysis of the Ciliary Number and Length

Primary cilia were identified by the juxtaposition of ARL13B signals (red) to centrin2 signals (green). To quantify the ciliary number and length, Z-stacked images from multiple fields were collected. The ciliary length was determined by tracing a line across the length of the primary cilium in a 3D Z-stack with ImageJ software [9]. Each representative ciliary region measured in tissues or organs consisted of at least 1000 cells (40× magnification). Ciliary length and orientation were assessed in a blinded fashion by two of the authors. The ciliated cells in each image were counted under a microscope. The cell numbers were determined by DAP nuclear staining. For muscle cells, we identified single cells by both DAPI and centrin2-GFP staining (usually, each cell has a pair of centrioles). Some cilia were oriented along the long axis of the nucleus. At least 30 images were assessed. For 3D reconstruction of cilia, Amira^®^ software was used (Template Graphics Software; Visage Imaging, San Diego, California, USA). To limit measurement error, for each cilium, the average of three measurements was used for statistical analysis. The percentage of ciliated cells was calculated from the ratio of ciliated cells to total cells observed in each sample (under 40× magnification; the analysis was performed with five sections collected from each sample). The organs and tissues of six mice were evaluated.

### 2.6. Time-Lapse Observations of Cilia in Living Nucleus Pulposus (NP) Tissue

For analysis of the NP, intervertebral discs were dissected between the fifth and sixth coccygeal vertebrae of 4-week-old ARL13B-mCherry; Centrin2-GFP cilium dual reporter-expressing transgenic mice and then immediately placed on slides for live imaging under a microscope. All images were visualized and videorecorded using a Leica DMI6000 inverted epifluorescence microscope (DMI6000B, Leica) with a Leica DFC365FX monochrome digital camera in conjunction with LAS-X acquisition software (Leica).

## 3. Results

### 3.1. Cilia Are Present in Different Tissues of the Respiratory and Circulatory Systems

Using the described genetic mouse model, we first confirmed that mCherry marks cilia in mouse tissues (Figures S1 and S2) via immunofluorescence staining of ARL13B and acetylated tubulin in tracheal tissue.

**Figure 1:**
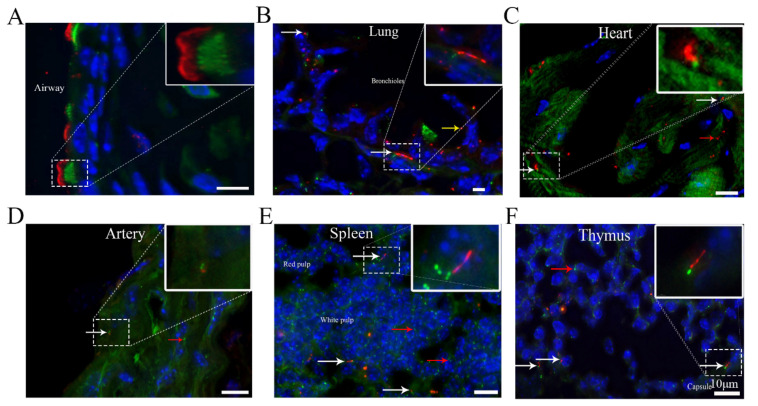
Cilia are present in different tissues of the respiratory system, circulatory system and immune system. (**A**) Trachea, (**B**) bronchioles, (**C**) heart, (**D**) artery, (**E**) spleen, (**F**) thymus. White arrow, cilium with an ARL13B-mCherry+ axoneme and Centrin2-GFP+ basal body. Yellow arrow, cell with only ARL13B-mCherry. Red arrow, cell with only Centrin2-GFP. Green, Centrin2-GFP. Red, ARL13B-mCherry. Blue, DAPI. Scale bar, 10 μm.

Then, we examined the patterns of centrioles and cilia in respiratory system components, including the trachea and bronchioles. In the trachea, consistent with previous observations [10], multiple mCherry+ cilia with GFP+ basal bodies were present on almost the entire airway epithelium. In tracheal cartilage, mCherry+ primary cilia were detected in 72.2 ± 1% of chondrocytes, and the length of primary cilia varied between 3 and 5 μm, consistent with the number and length of primary cilia in chondrocytes of articular cartilage [11] (Figure 1A) (Table 1). In bronchioles, mCherry+ cilia with GFP+ centrioles were detected on the entire epithelium (Figure 1B). Moreover, 24.7 ± 1.6% of smooth muscle cells surrounding the bronchiolar epithelium presented mCherry+ primary cilia.

Primary cilia have been reported to be present in the heart and arteries in a previous study [12,13]. We found that the frequency of mCherry+ cilia in myocytes was 8.7 ± 0.6%, and the ciliary length ranged from 1 to 3 μm. Most interestingly, small puncta of ARL13B-mCherry+ accumulated in some myocytes (Figure 1C). In the arteries, the frequency of ciliated endothelial cells was 6 ± 0.1%, and the ciliary length ranged from 0.5 to 2 μm. The profiles of primary ciliary axonemes in endothelial cells were parallel along the long axis of the cell and projected into the surrounding extracellular matrix (ECM) at a range of angles.

### 3.2. Primary Cilia Are Present in Different Organs of the Immune System

The spleen contains two types of tissues: white pulp and red pulp. Red pulp is composed of venous sinuses and splenic cords. White pulp mostly consists of immune cells [14]. The thymus is an immune organ in humans that produces dendritic cells, macrophages, and mature thymocytes. It has been reported that lymphocytes are one of the few cell types that do not possess cilia [15]. Consistent with this finding, we found that the majority of cells in the spleen and thymus did not have ARL13B-mCherry+ cilia. However, unexpectedly, we newly discovered that mCherry+ primary cilia existed in both spleen cells and thymocytes. The frequency of primary cilia in spleen cells was 13.4 ± 0.9%, while it was 20.3 ± 0.9% in thymus cells. In addition, the length of the primary cilia was 3–6 μm in the spleen and thymus (Figure 1E,F) (Table 1). It will be interesting to further investigate the functions of these cilia in specific immune cell types and in the immune system as a whole.

### 3.3. Cilia Are Present in Different Organs of the Digestive System, Including Hepatocytes

**Figure 2:**
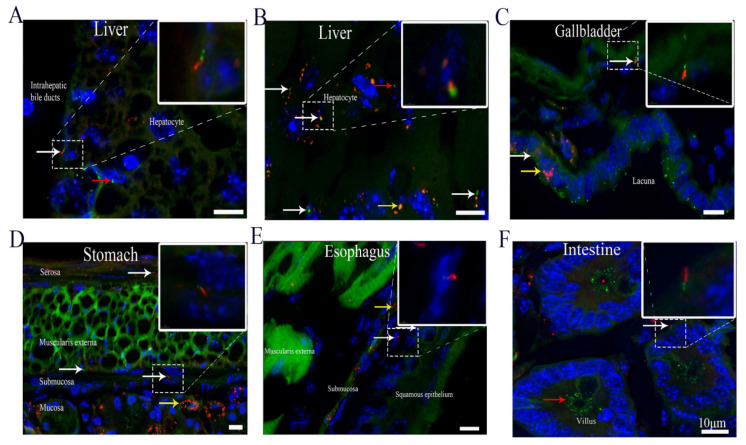
Cilia are present in different organs of the digestive system. (**A**,**B**) Hepatocytes and intrahepatic bile ducts in the liver, (**C**) gallbladder, (**D**) stomach, (**E**) esophagus, and (**F**) intestine. White arrow, cilium with an ARL13B-mCherry+ axoneme and Centrin2-GFP+ basal body. Yellow arrow, cell with only ARL13B-mCherry. Red arrow, cell with only with Centrin2-GFP. Green, Centrin2-GFP. Red, ARL13B-mCherry. Blue, DAPI. Scale bar, 10 μm.

It has been reported that no primary cilia are present on hepatocytes [16]. Hepatocytes are polygonal in shape, and their cell membrane can come in contact with either sinusoids (on the sinusoidal face) or neighboring hepatocytes (on the lateral faces) [17]. Our morphological analysis revealed that 8.2 ± 0.3% of hepatocytes had mCherry+ primary cilia and that the ciliary length was 0.5–2 μm. Interestingly, small puncta of ARL13B-mCherry+ particles were detected in some hepatocytes (Figure 2A,B). Cholangiocytes are epithelial cells in the bile duct, and primary cilia in these cells have been reported to extend from the apical plasma membranes into the ductal lumen [16]. Consistent with the findings of previous studies [16], the frequency of ciliated cells in our study was 43.5 ± 2%, and the length of the primary cilia ranged from 5 to 7 μm (Figure 2A). In the gallbladder, ARL13B-mCherry+ primary cilia were present on 16.1 ± 0.8% of epithelial cells and 9.1 ± 0.4% of smooth muscle cells (Figure 2C). The primary ciliary length ranged from 1.5 to 3 μm in the epithelial cells and 3 to 5 μm in the smooth muscle cells (Table 1). Notably, many cells in the gallbladder epithelium had no cilia but displayed Centrin2-GFP-labeled centrioles on the apical cell surface. Accumulation of ARL13B-mCherry+ particles was observed in gallbladder epithelial cells (Figure 2C). Primary cilia in the gastric epithelium have been reported previously [18]. Structurally, the stomach wall is composed of four layers. We found that each layer had some ciliated cells. The ciliary length ranged from 0.5 to 3 μm in the mucosa and muscularis externa and from 0.5 to 5 μm in the submucosa and serosa. Primary cilia were detected in 5.9 ± 0.2%, 14 ± 0.7%, 9.1 ± 0.3%, and 21.2 ± 0.6% of the cells in the mucosa, submucosa, muscularis externa, and serosa, respectively (Table 1). Interestingly, numerous ARL13B-mCherry+ particles were deposited in some epithelial cells and formed a well-organized pattern in the mucosal layer (Figure 2D). To the best of our knowledge, cilia in the esophagus have never been investigated. We found that the ciliary length ranged from 3 to 5 μm in squamous epithelial cells and from 0.5 to 3 μm in the submucosa and muscularis externa cells (Figure 2E). Primary cilia were detected in 7 ± 0.1%, 7.4 ± 0.1%, and 9 ± 0.2% of squamous epithelial cells, submucosal cells and muscularis externa cells, respectively (Table 1). Additionally, accumulation of ARL13B-mCherry+ particles was observed in some submucosal cells (Figure 2E). The intestine plays an essential role by absorbing water, vitamins, and electrolytes from waste material [19]. Interestingly, similar to the pattern in gallbladder epithelial cells, a large number of Centrin2-GFP+ particles were located on the apical cell surface in the intestinal epithelium (Figure 2F).

### 3.4. Cilia Are Present in Different Organs in the Urinary and Reproductive System

**Figure 3:**
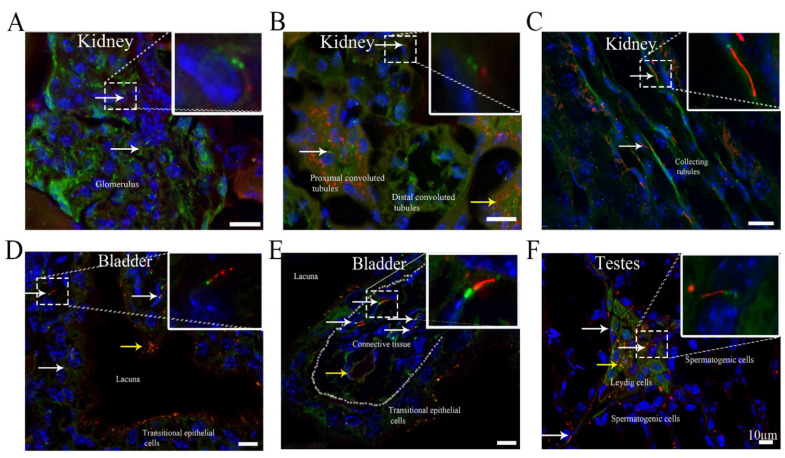
Cilia are present in different organs in the urinary and reproductive system. (**A**) Kidney glomerulus, (**B**) kidney proximal convoluted tubules and distal convoluted tubules, (**C**) kidney collecting tubules, (**D**,**E**) bladder, and (**F**) testes. White arrow, cilium with an ARL13B-mCherry+ axoneme and Centrin2-GFP+ basal body. Yellow arrow, cell with only ARL13B-mCherry. Green, Centrin2-GFP. Red, ARL13B-mCherry. Blue, DAPI. Scale bar, 10 μm.

Although primary cilia in the kidney have been extensively investigated [20], the distributions and features of cilia in the kidney remain unclear. Our results showed that ARL13B-mCherry+ cilia were present and that the ciliary length ranged from 1.5 to 3 μm in the glomerulus, proximal convoluted tubules and distal convoluted tubules and from 10 to 15 μm in the collecting tubules. Primary cilia were observed in 9.6 ± 0.4%, 23.9 ± 0.6%, 12.6 ± 0.2%, and 54.5 ± 3.8% of the cells in the glomerulus, proximal convoluted tubules, distal collecting tubules, and collecting tubules, respectively (Figure 3A–C) (Table 1). The epithelium in proximal convoluted tubules and distal convoluted tubules exhibited shorter cilia with an average ciliary length of 0.5–3 μm (Figure 3B). The proximal tubule consists of polarized monolayer cells, which are characterized by a brush border [21]. Through morphological analysis [21], we found large quantities of ARL13B-mCherry+ signals with a well-organized pattern (with cilia facing the tubule lumens) in proximal convoluted tubules (Figure 3B), suggesting that cilia may have a function in secretion or absorption. The cilia in collecting tubules were much longer than those in proximal tubules and were well oriented in one direction (Figure 3C). The collecting tubules transport urine and absorb water in the kidneys. Few studies have investigated cilia on bladder epithelial cells [22]. Approximately 17.4 ± 0.7% of mCherry-expressing primary cilia (Figure 3D) were observed in transitional epithelial cells, and the length of the primary cilia was 1–3 μm. Many ARL13B-mCherry+ particles were present in the transitional epithelial cells, which were near the lacunae (Figure 3D). ARL13B-mCherry+ primary cilia were found in 31.8 ± 0.8% of the cells in the musculoskeletals underneath the epithelium, and the ciliary length varied from 5 to 7 μm (Figure 3D,E).

Cilia have been reported to be present in testes in several studies [23,24]. We found that both primary cilia and ARL13B-mCherry+ particles were present in Leydig cells. The percentages of primary ciliated cells were 23 ± 1.3% among Leydig cells and 10.9 ± 1% among spermatogenic cells, and the ciliary length was 3–10 μm in the Leydig cells and 0.5–2 μm in the spermatogenic cells. Most of the primary ciliary axonemes in the Leydig cells were parallel to the long axis of the nucleus (Figure 3F).

### 3.5. Ciliary Arrangement and Patterns in the Cerebral Cortex of the Brain

**Figure 4:**
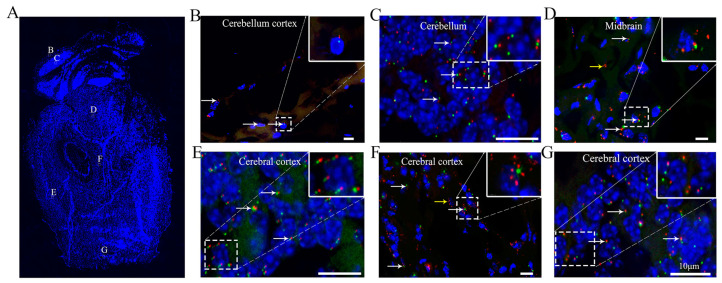
The cilia in the cerebral cortex are well, pointing to the center of the brain. (**A**) Sagittal sections of the brain, (**B**) cerebellar cortex, (**C**) cerebellum, (**D**) midbrain, and (**E**–**G**) cerebral cortex. Many ARL13B+ particles are visible in (**F**). White arrow, cilium with axoneme and basal body. Yellow arrow, localization of ARL13B. Red arrow, cells with only Centrin2. Green, Centrin2-GFP. Red, ARL13B-mCherry. Blue, DAPI. Scale bar, 10 μm.

It has been reported that the mean ciliary length varies across brain regions, ranging from 2.1 to 9.4 μm across 23 regions of the central nervous system [25]. Our results showed various ciliary lengths in different regions (Figure 4A). ARL13B-mCherry+ primary cilia were present in 19.3 ± 0.8%, 73.9 ± 3.7%, 9.6 ± 0.1%, and 91.7 ± 3.8% of the cells in the mouse cerebellar cortex (Figure 4B), cerebellum (Figure 4C), midbrain (Figure 4D), and cerebral cortex (Figure 4E–G), respectively, and the ciliary lengths varied from 0.5 to 3 μm (Table 1). Cilia were short in the midbrain and cerebral cortex, but some mCherry+ particles were detected in the cytoplasm of the midbrain (Figure 4D). Most interestingly, the cilia in the some regions (Figure 4E,G) of the cerebral cortex were well oriented, with all the cilia pointing to the middle of the brain. This is the first study to show the specific orientation of cilia in the parenchyma, which may suggest a novel function of cilia in the brain that has not yet been elucidated. It will be interesting to investigate and identify the cell type (neurons or glial cells) associated with these featured cilia and to determine the functions of these cilia in the brain.

### 3.6. Cilia Are Present in Different Organs in the Musculoskeletal System

**Figure 5:**
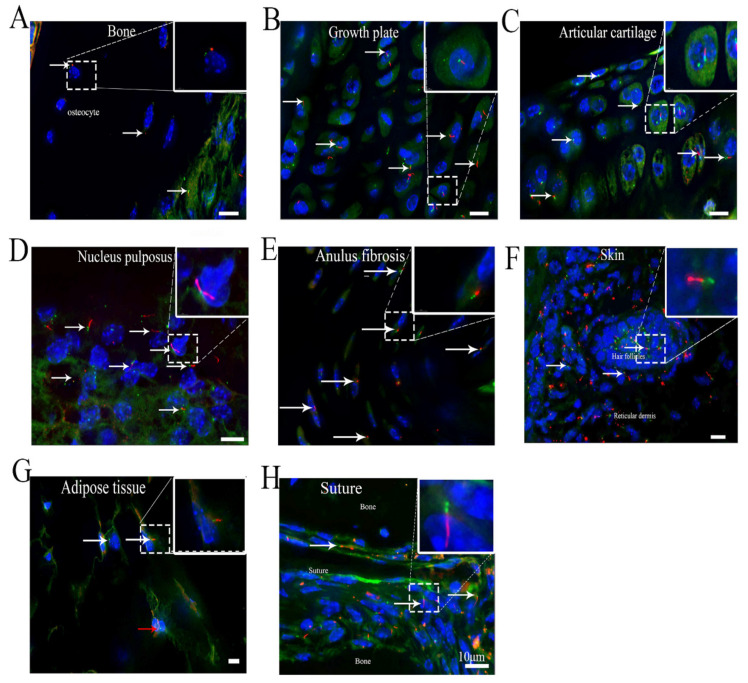
Cilia are present in different organs in the musculoskeletal system. (**A**) Bone tissue from femur, (**B**) growth plate from tibia, (**C**) articular cartilage, (**D**) NP in intervertebral disc, (**E**) AF in intervertebral disc, (**F**) skin, (**G**) adipose tissue, and (**H**) suture. White arrow, cilium with an axoneme and basal body. Yellow arrow, localization of ARL13B. Red arrow, cell with only Centrin2. Green, Centrin2-GFP. Red, ARL13B-mCherry. Blue, DAPI. Scale bar, 10 μm.

Osteoblasts, the main bone-forming cells, are derived from mesenchymal progenitors, and are characterized by the production of alkaline phosphatase, osteocalcin, and type I collagen. A small subset of osteoblasts are trapped in the bone matrix to become osteocytes. Consistent with our previous studies in which an antibody against acetylated tubulin was used for cilium detection, the length of ARL13B-mCherry+ primary cilia varied from 3 to 3.5 μm in osteoblasts and 2.5 to 3 μm in osteocytes [26]. The percentage of primary ciliated cells was 86.8 ± 3% in osteoblasts and 81 ± 2.9% in osteocytes (Table 1). Most ciliary axonemes in osteoblasts and osteocytes were parallel to the long axis of the nucleus (Figure 5A).

Primary cilia in chondrocytes have been investigated by many studies [11,27]. In this study, ARL13B-mCherry+ primary cilia were detected in 68.9 ± 4.1% of growth plate chondrocytes and 49.6 ± 1.5% of articular cartilage chondrocytes. The ciliary length ranged from 2 to 4 μm (Table 1). The orientation of the primary cilia in chondrocytes has also been investigated previously [11]. Through our observations, some ciliary axonemes in articular cartilage chondrocytes were observed to be parallel to the long axis of the nucleus (Figure 5B,C).

In intervertebral discs, the length of ARL13B-mCherry+ primary cilia was 0.5–15 μm in the nucleus pulposus (NP) and 0.5–3.5 μm in the annulus fibrosis (AF). Approximately 33.62 ± 0.4% of NP cells and 36.1 ± 1.9% of AF cells were ciliated in the third and fourth lumbar intervertebral discs of the mice (Figure 5D,E). The NP is composed of a proteoglycan-and-water gel that is loosely held together by an irregular network of type II collagen and elastin fibers. Recent studies have suggested that NP cells are derived from the embryonic node and notochord [28]. To analyze whether cilia in NP cells in the postnatal stage can move similarly to those in embryonic notochord cells, we isolated whole NPs from intervertebral discs between the fifth and sixth coccygeal vertebrae in the tail and observed and recorded live images of ciliary movement with a microscope. Surprisingly, approximately 2% of the cilia showed irregular movement in the NP extracellular fluid, which is different from the clockwise movement of primary cilia in embryonic notochord cells [29] (supplementary Videos S1 and S2). Interestingly, we observed that the primary cilia in the AF were located on the inner sides of the cells and were always oriented in one direction parallel to the long axis of the nucleus (Figure 5E).

In the skin, approximately 33.4 ± 1.1% of hair follicle cells and 52.8 ± 0.9% of reticular dermal cells were ciliated (Figure 5F). The ciliary length in the hair follicles and reticular dermis varied from 1.5 to 5 μm. ARL13B-mCherry+ primary cilia were present on 18.7 ± 0.5% of adipocytes in adipose tissues, and the ciliary length ranged from 3 to 6 μm, which is consistent with previous observations [30] (Figure 5G) (Table 1).

The junctures between the bones of the skull where the bones are held tightly together by fibrous tissue are called sutures. It has been reported that sutures are important for calvarial bone development and maintenance [31]. Approximately 85.5 ± 1.1% of the cells in the sutures possessed ARL13B-mCherry+ primary cilia, and the ciliary length varied from 5 to 7 μm (Figure 5H) (Table 1).

### 3.7. ARL13B+ Inclusions Are Present in the Thyroid and Islets of Langerhans

**Figure 6:**
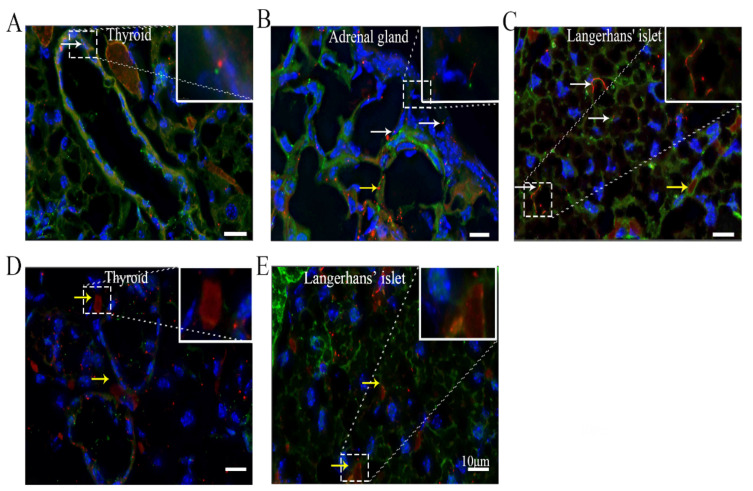
ARL13B+ inclusions are present in the thyroid and islets of Langerhans. (**A**) Thyroid, (**B**) adrenal gland, (**C**) islets of Langerhans, (**D**) thyroid cells with ARL13B-mCherry+ inclusions (**E**) cells in islets of Langerhans with ARL13B-mCherry+ inclusions. White arrow, cilium with an axoneme and basal body. Yellow arrow, cell with ARL13B-mCherry+ inclusions or particles. Blue, DAPI. Green, Centrin2-GFP. Red, ARL13B-mCherry. Scale bar, 10 μm.

Given that cilia project into the extracellular environment and have the ability to concentrate signaling cascade proteins in the ciliary compartment and membrane, primary cilia are thought to regulate endocrine pathways [32]. However, the features and functions of cilia in the endocrine system are largely unknown. Our results showed that approximately 23.9 ± 2.2% of thyroid epithelial cells possessed ARL13B-mCherry+ primary cilia, and the ciliary length varied from 0.5 to 2.5 μm [33] (Figure 6A) (Table 1). ARL13B-mCherry+ primary cilia were present on 7.3 ± 1.2% of adrenal gland epithelial cells, and the ciliary length varied from 3 to 7 μm [34]. Some ARL13B-mCherry+ particles also accumulated in areas of the adrenal gland epithelium (Figure 6B). The percentage of ARL13B-mCherry+ primary cilia in the islets of Langerhans in the pancreas was 21.1 ± 1.6%, and the ciliary length varied from 5.5 to 15 μm [35] (Figure 6C). Most interestingly, ARL13B-mCherry+ inclusions were observed in some cells of the thyroid colloid (Figure 6D) and islet (Figure 6E).

## 4. Discussion

We identified cilia in different organs and tissues by using ARL13B-mCherry; Centrin2-GFP cilium dual reporter-expressing transgenic mice. This study reveals the existence and distribution of cilia and centrioles in different tissues and organs and provides new insights for further comprehensive study of ciliary function in these organs and tissues.

Interestingly, we found that a few motile cilia in NP cells of intervertebral discs moved around the extracellular fluid in an irregular way. The NP is reportedly derived from the embryonic node and notochord. Cilia in the embryonic node have attracted the interest of developmental biologists, who have hypothesized that leftward-directed fluid flow is essential for left-right (LR) axis determination in mice. In agreement with this hypothesis, we found that approximately 2% of cilia showed irregular movement in NP extracellular fluid; such movement is different from the clockwise movement of primary cilia in embryonic notochord cells. NP is a hydrated, gel-like structure, which enable movement of the spine via deformation and alteration of the shape of the intervertebral disc during compression, tilting, and twisting motions [36]. Our previous study showed that ciliary length varied widely in the NP, ranging from 0.5 to 15 μm, and that eliminating cilia from NP cells caused the gel-like matrix in the NP to become less compact and to be markedly reduced [37]. In the epithelium of the airway, multiciliate cells have synchronized cilia that beat to move mucus over the epithelial surface [38]. In the brain, ‘ependymal flow’ of cerebrospinal fluid (CSF) is generated by multi-ciliated cells lining the ventricles that move signaling molecules through the central nervous system and are essential for the migration of young neurons produced in adult subventricular tissues [39]. In the urinary and reproductive system, the single flagellum of a sperm may propel the whole cell body through the surrounding liquid [40]. However, the function of motile cilia in the NP is unclear, and further investigation is needed.

It has previously been reported that cilia are undetectable on hepatocytes [16]. Using a cilium dual reporter-expressing mouse model, we found that a small group of hepatocytes were ciliated. Among ciliopathies, liver cysts and fibrosis are common pathological changes, suggesting that primary cilia are essential to the normal function of the liver [41].

The gallbladder, intestine, and thyroid epithelia play important roles in secretion and absorption. Recently, the centrosomes of natural killer (NK) and invariant NK T (iNKT) cells (cytolytic cells of the innate immune system) have been reported to play important roles in the direct secretion of lysosomes into immunological synapses [42,43], suggesting that centrosomes may be key participants in cell secretion. In support of centriole importance, we observed many Centrin2-GFP-labeled centrioles without cilia in the epithelia of the gallbladder, intestine, and thyroid. The similar features and distributions of cilia and Centrin2 in organs with robust secretion and absorption functions indicate that centrioles may play important roles in secretion independent of cilia.

ARL13B has been reported to localize specifically to cilia and is regarded as a marker for cilia [2]. Our results revealed that ARL13B-mCherry+ particles were present in the esophageal submucosa, kidney proximal convoluted tubule epithelium, thyroid, islets, bladder transitional epithelium, and some cells in the brain. These findings suggest that ARL13B may play an important role in cell or organ function independent of cilia [44]. Primary cilia in neurons have often been regarded as rare vestigial curiosities [22]. However, neuronal cilia are now gaining recognition as ubiquitous organelles in the mammalian brain, raising speculation about ciliary functions. Several markers have been used to stain cilia in different regions of the brain, and the characteristics of cilia have been reported to vary in different brain regions [45]. Surprisingly, we found, for the first time, that the ARL13B-mCherry+ neuronal cilia in the cerebral cortex were well oriented toward the center of the brain (supplementary Figure S3). The central nervous system develops from a neural tube that grows from a single layer of neural progenitor cells. The patterning or formation of distinct regions of the central nervous system is achieved through progressive divisions along the dorsoventral and rostrocaudal axes of neural progenitor domains. Morphogens secreted by discrete populations of cells form concentration gradients along those axes, and the gradients specify different fates of neural progenitors, thereby patterning the central nervous system [46]. A hypomorphic mutation in the cilium Ift88 gene can cause severe disorganization of telencephalic structures, resulting in malformed dorsomedial structures (cortical hem, hippocampal primordium, and choroid plexus), incomplete divisions between the dorsal and ventral (pallial and subpallial) forebrain, and between the telencephalon and diencephalon, and the formation of rosette-shaped heterotopias with central lumens. These phenotypes are highly similar to those observed in Hh gene-mutant mice [46]. As signal transduction centers (especially for Hh signaling), well-oriented neuronal cilia may play important roles during signaling gradient formation or brain pattern maintenance.

Cilia are regarded as secretory organelles in many tissues [47]. Endocrine organs, including the islets and thyroid, have robust secretion functions. However, few studies have reported cilia in these organs [34]. Our results showed that some ARL13B-mCherry+ primary cilia were present in the islets of Langerhans and that the ciliary length varied from 10 to 15 μm. These results suggest that cilia may play important roles in pancreatic function. One study has supported this idea, revealing that cilia loss can always be detected in diabetes patients [48]. Our recent study also confirmed that diabetes induces cilia loss in all ciliated cells [27]. Moreover, ARL13B-mCherry+ inclusions were observed in some tubules of islets and the thyroid colloid, indicating that the ARL13B protein can be secreted by cells. Notably, ciliopathy is diagnosed by whole-exome sequencing, which is an expensive and time-consuming process [49]. Further study is needed to determine whether the serum levels of cilium-related proteins, such as ARL13B, could be detected to diagnose ciliopathy in the future.

## 5. Conclusions

In summary, our study reveals new information regarding cilia, centrioles, and the distributions and orientations of these organelles and suggests the possible secretion of particles from different organs and tissues in a mouse system. The findings of this study lay a foundation facilitating further studies on the roles and functions of cilia in different organs and provide insights for the development of new diagnostic and therapeutic strategies for Joubert syndrome and other ciliopathies.

## Figures and Tables

**Table 1:** The cilia in each organ or tissues.

		Motile Cilia(%)	Primary Cilia(%)	Ciliary Length (μm)	Orientation
Trachea	Epithelium	100%	-	5.0–7.0	Point to airway
	Cartilage	-	72.2 ± 1	3.0–5.0	N/A
Bronchioles	Epithelia	100%	-	5.0–7.0	Pointing to airway
	Smooth muscle	-	24.7 ± 1.6	3.0–5.0	N/A
	Heart	-	8.7 ± 0.6	1.0–3.0	N/A
Arteries	Endothelium	-	6 ± 0.1	0.5–2.0	The long axis of epithelia nuclear
Immune system	Spleen	-	13.4 ± 0.9	3.0–6.0	N/A
	Thymus	-	20.3 ± 0.9	3.0–6.0	N/A
Liver	Hepatocytes	-	8.2 ± 0.3	0.5–2.0	N/A
	Cholangiocytes	-	43.5 ± 2	5.0–7.0	N/A
	Intestine	-	9.8 ± 0.6	3.0–5.0	N/A
Gallbladder	Epithelia	-	16.1 ± 0.8	1.5–3.0	N/A
	Smooth muscle	-	9.1 ± 0.4	3.0–5.0	The long axis of nuclear
Stomach	Mucosa	-	5.9 ± 0.2	0.5–3.0	N/A
	Submucosa	-	14 ± 0.7	3.0–5.0	N/A
	Muscularis externa	-	9.1 ± 0.3	0.5–3.0	N/A
	Serosa	-	21.2 ± 0.6	3.0–5.0	The long axis of nuclear
Esophagus	Squamous epithelium	-	7 ± 0.1	3.0–5.0	The long axis of epithelia nuclear
	Submucosa	-	7.4 ± 0.1	0.5–3.0	The long axis of nuclear
	Muscularis externa	-	9 ± 0.2	0.5–3.0	N/A
Kidneys	Glomeruli in kidney	-	9.6 ± 0.4	1.5–3.0	N/A
	Proximal convoluted tubules	-	23.9 ± 0.6	1.5–3.0	Toward the lumen of the tubules
	Distal convoluted tubules	-	12.6 ± 0.2	1.5–3.0	N/A
	Collecting tubules	-	54.5 ± 3.8	10.0–5.0	Same direction as fluid
Bladder	Connective tissues	-	31.8 ± 0.8	5.0–7.0	N/A
Testes	Leyding cells	-	23.0 ± 1.3	3.0–10.0	N/A
	Spermatogenic cells	-	10.9 ± 1	0.5–2.0	N/A
Bone	Osteoblasts	-	86.8 ± 3	3.0–3.5	Long axis of nuclear
	Osteocytes	-	81 ± 2.9	2.5–3.0	Long axis of nuclear
Cartilage	Growth plate	-	68.9 ± 4.1	2.0–4	Long axis of the growth plate chondrocyte
	Artcular cartilage	-	49.6 ± 1.5	2.0–4.0	Long axis of the artcular cartilage chondrocyte
Intervertebral discs	Nucleus pulposus	-	33.2 ± 0.4	0.5–15.0	N/A
	Annulus fibrosus	-	36.1 ± 1.9	0.5–3.5	Aligned parallel to the long axis of the aunulus fibrosus cells
Skin	Hair follicles	-	33.4 ± 1.1	1.5–5.0	N/A
	Reticular dermis		52.8 ± 0.9	1.5–5.0	N/A
	Adipose tissue	-	18.7 ± 0.5	3.0–6.0	N/A
	Sutures	-	85.5 ± 1.1	5.0–7.0	Long axis of the nuclear
Brain	Cerebellum cortex	-	19.3 ± 0.8	0.5–3.0	N/A
	Cerebellum	-	73.9 ± 3.7	0.5–3.0	N/A
	Midbrain	-	9.6 ± 0.1	0.5–3.0	N/A
	Cerebral cortex	-	91.7 ± 3.8	0.5–3.0	Pointing to centre of brain
Endocrine system	Thyroid	-	23.9 ± 2.2	0.5–2.5	N/A
	Adrenal gland	-	7.3 ± 1.2	3.0–7.0	N/A
	Islet of Langerhans	-	21.1 ± 1.6	5.5–15.0	N/A

## Data Availability

The data are contained within the article or supplementary material.

## Supplementary Materials

The following are available online at https://www.mdpi.com/article/10.3390/cells10071623/s1, Figure S1: Antibody costaining of ARL13B and DAPI confirmed the specific targeting of cilia in ARL13B-mCherry; Centrin2-GFP mouse tracheal tissue, Figure S2: Immunostaining for acetylated tubulin in the trachea confirmed that ARL13B is a reliable marker for cilia in the ARL13B-mCherry; Centrin2-GFP mouse model, Figure S3: Schematic diagram of cilium distribution in the mouse brain, Vedio S1: The representative vedio to show the cilia rotation in nucleus puplposus tissues, Vedio S2: The representative vedio to show the cilia rotation in nucleus puplposus tissues.
