# Supplementary material for: Identification of Cilia in Different Mouse Tissues
Source: Cells. 2021 Jun 29;10(7):1623. doi: 10.3390/cells10071623 (PMC8307782; doi:10.3390/cells10071623)
Supplement: Supplementary file 1 [file cells-10-01623-s001.zip › Supplementary.pdf]

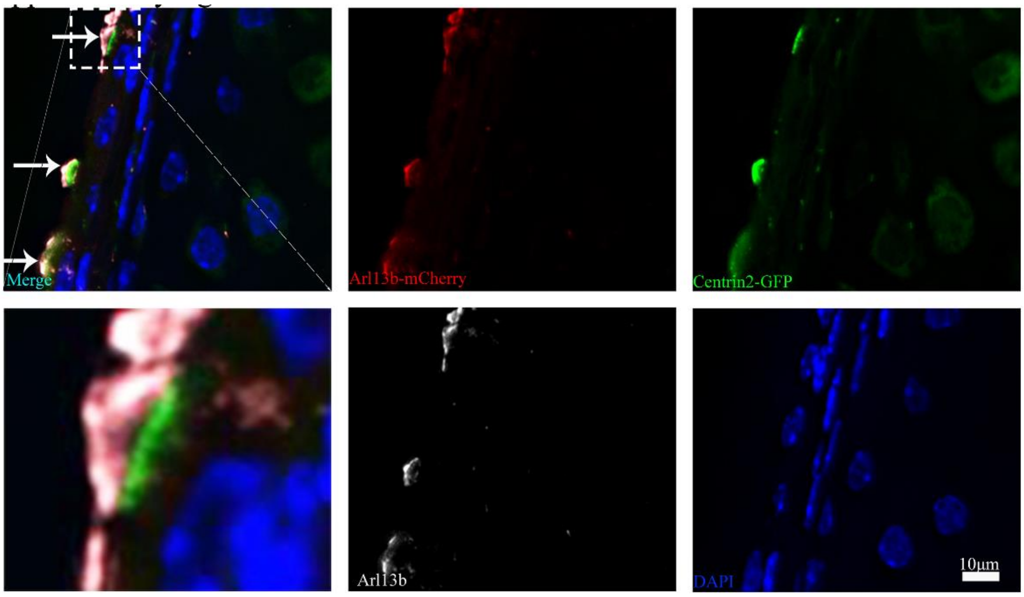

Figure S1. Antibody costaining of ARL13B and DAPI confirmed the specific targeting of cilia in ARL13B-mCherry;Centrin2-GFP mouse tracheal tissue. White arrows point to motile cilia. Blue, DAPI. Green, Centrin2-GFP. Red, ARL13B-mCherry. White, ARL13B antibody staining. Scale bar, 10 μm.

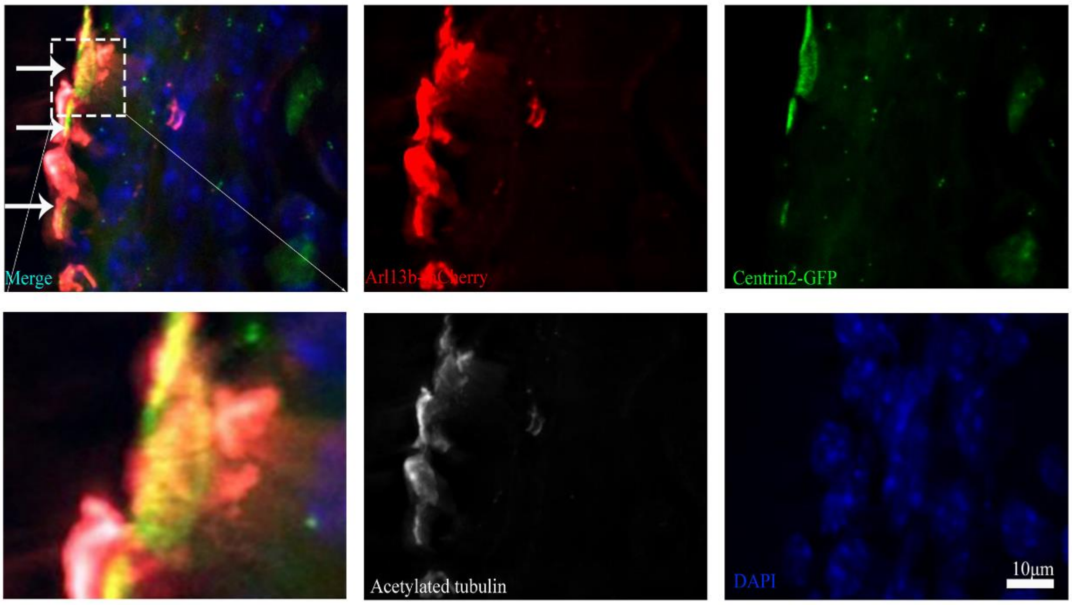

Figure S2. Immunostaining for acetylated tubulin in the trachea confirmed that ARL13B is a reliable marker for cilia in the ARL13B-mCherry;Centrin2-GFP mouse model. *White arrows point to motile cilia. Blue, DAPI. Green, Centrin2-GFP. Red, ARL13B-mCherry. White, acetylated tubulin antibody staining. Scale bar, 10 μm.*

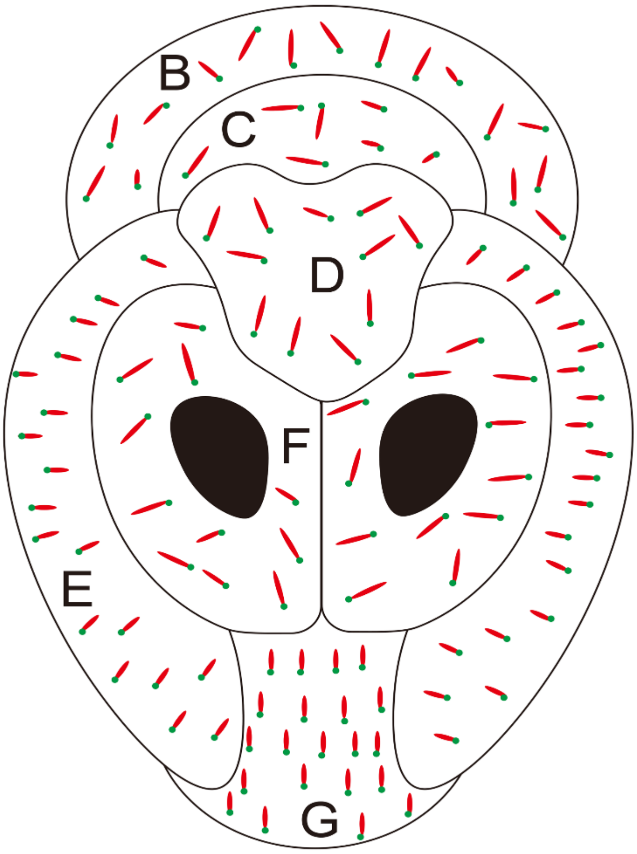

Figure S3. Schematic diagram of cilium distribution in the mouse brain. (**A**) Sagittal sections of the brain, (**B**) cerebellar cortex, (**C**) cerebellum, (**D**) midbrain, and (**E–G**) cerebral cortex. Green, Centrin2-GFP. Red, ARL13B-mCherry.

Vedio S1 The representative vedio to show the cilia rotation in nucleus puplposus tissues

Bule DAPI. Red, ARL13B-mCherry.

Vedio S2 The representative vedio to show the cilia rotation in nucleus puplposus tissues

Red, ARL13B-mCherry.
