# Supplementary figures and images for: Identification of Cilia in Different Mouse Tissues
Source: Cells. 2021 Jun 29;10(7):1623. doi: 10.3390/cells10071623 (PMC8307782; doi:10.3390/cells10071623)

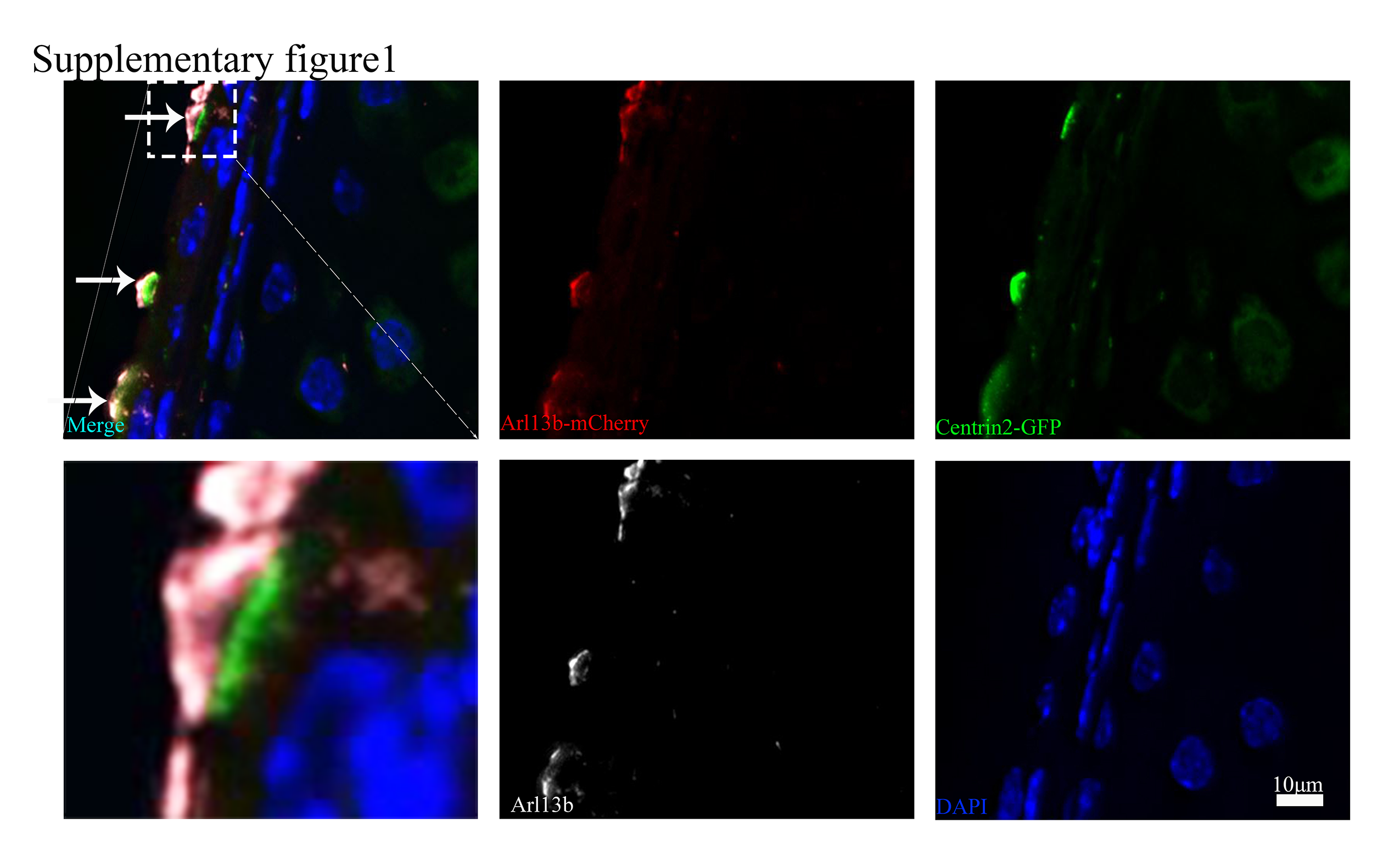

Supplement: Supplementary file 1 [file cells-10-01623-s001.zip › Supplementary figure1.tif]

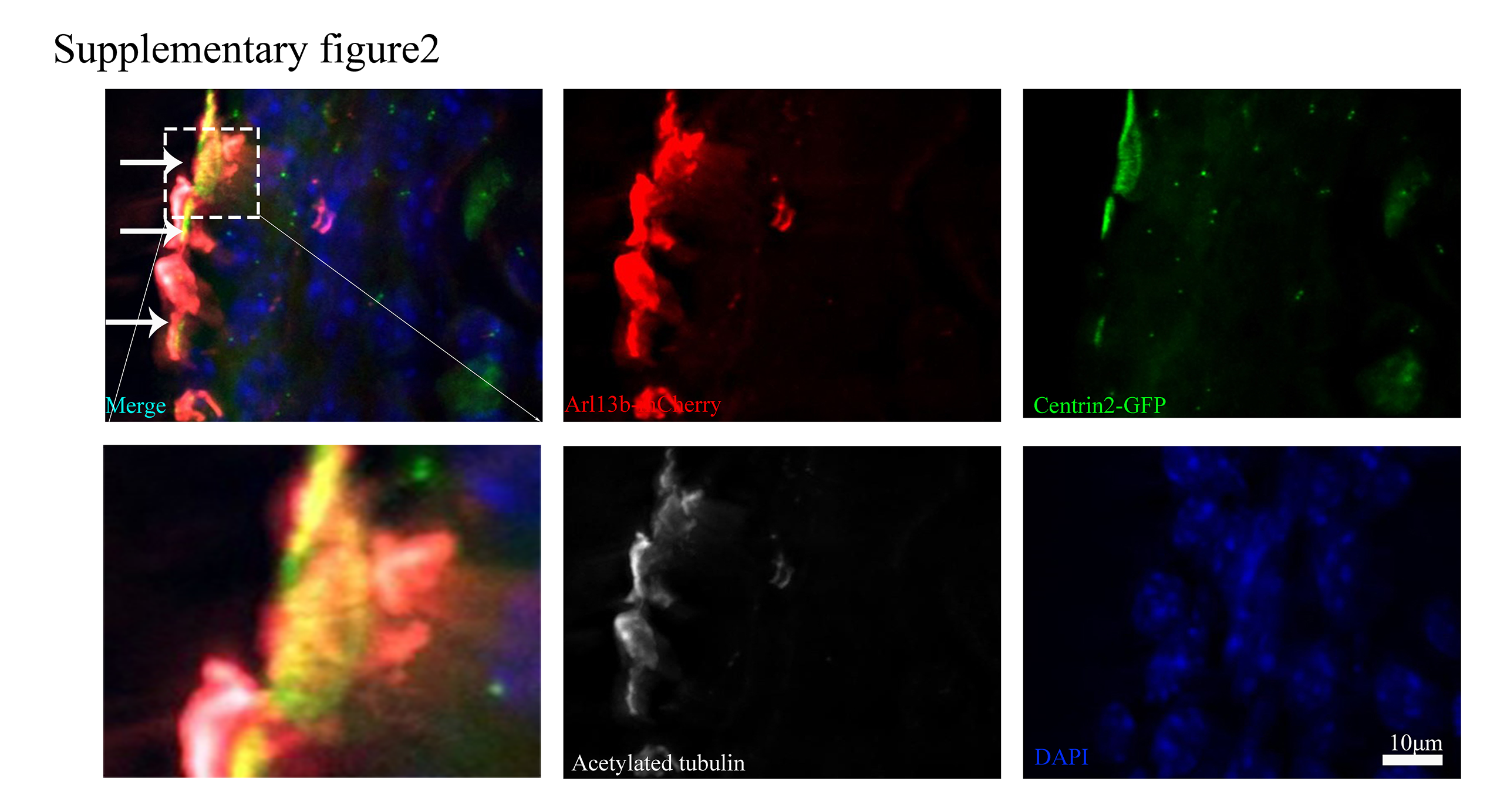

Supplement: Supplementary file 1 [file cells-10-01623-s001.zip › Supplementary figure2.tif]

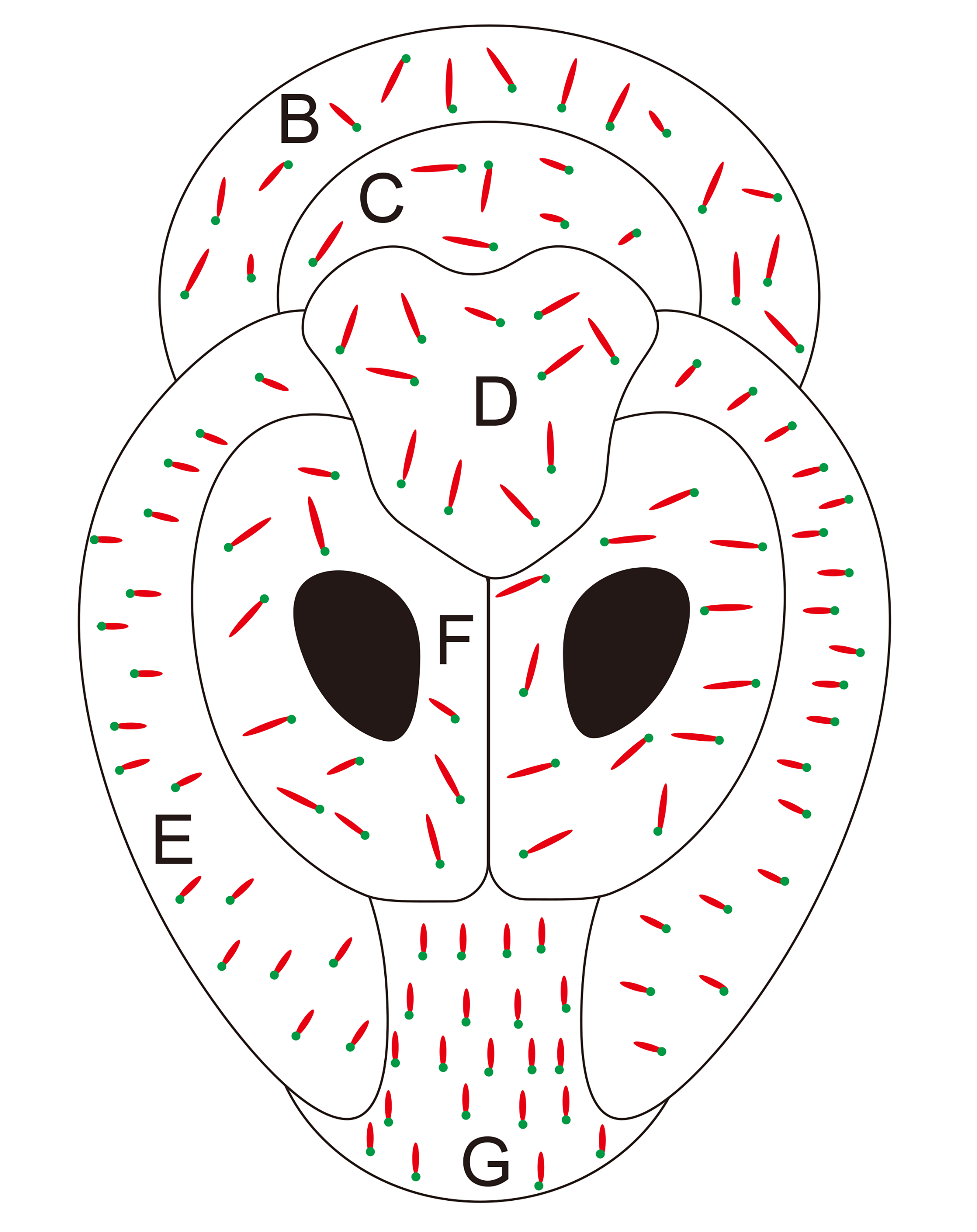

Supplement: Supplementary file 1 [file cells-10-01623-s001.zip › Supplementary figure3.tif]
